# Label-Free Oligonucleotide-Based SPR Biosensor for the Detection of the Gene Mutation Causing Prothrombin-Related Thrombophilia

**DOI:** 10.3390/s20216240

**Published:** 2020-10-31

**Authors:** Rodrigo Sierpe, Marcelo J. Kogan, Soledad Bollo

**Affiliations:** 1 Laboratorio de Biosensores, Facultad de Ciencias Químicas y Farmacéuticas, Universidad de Chile; Santos Dumont N° 964, Independencia, Santiago 8380492, Chile; rsierpe@ciq.uchile.cl; 2 Laboratorio de Nanobiotecnología y Nanotoxicología, Facultad de Ciencias Químicas y Farmacéuticas, Universidad de Chile; Santos Dumont N° 964, Independencia, Santiago 8380492, Chile; mkogan@ciq.uchile.cl; 3 Advanced Center for Chronic Diseases (ACCDiS), Universidad de Chile and Pontificia Universidad Católica de Chile, Santiago 8380492, Chile

**Keywords:** surface plasmon resonance, biosensors, oligonucleotides, thrombophilia

## Abstract

Prothrombin-related thrombophilia is a genetic disorder produced by a substitution of a single DNA base pair, replacing guanine with adenine, and is detected mainly by polymerase chain reaction (PCR). A suitable alternative that could detect the single point mutation without requiring sample amplification is the surface plasmon resonance (SPR) technique. SPR biosensors are of great interest: they offer a platform to monitor biomolecular interactions, are highly selective, and enable rapid analysis in real time. Oligonucleotide-based SPR biosensors can be used to differentiate complementary sequences from partially complementary or noncomplementary strands. In this work, a glass chip covered with an ultrathin (50 nm) gold film was modified with oligonucleotide strands complementary to the mutated or normal (nonmutated) DNA responsible for prothrombin-related thrombophilia, forming two detection platforms called mutated thrombophilia (MT) biosensor and normal thrombophilia (NT) biosensor. The results show that the hybridization response is obtained in 30 min, label free and with high reproducibility. The sensitivity obtained in both systems was approximately 4 ΔμRIU/nM. The dissociation constant and limits of detection calculated were 12.2 nM and 20 pM (3 fmol), respectively, for the MT biosensor, and 8.5 nM and 30 pM (4.5 fmol) for the NT biosensor. The two biosensors selectively recognize their complementary strand (mutated or normal) in buffer solution. In addition, each platform can be reused up to 24 times when the surface is regenerated with HCl. This work contributes to the design of the first SPR biosensor for the detection of prothrombin-related thrombophilia based on oligonucleotides with single point mutations, label-free and without the need to apply an amplification method.

## 1. Introduction

Thrombophilia corresponds to an abnormality in coagulation or in the fibrinolytic system that increases the risk of coagulation leading to a thrombotic event. Deep thrombosis has an incidence of 0.1% (1 in 1000 people), and its frequency increases with advancing age [1]. Thrombophilia can be due to genetic or acquired factors, and the second most frequent factor corresponds to the mutation of the prothrombin gene G202210A, called prothrombin-related thrombophilia (PRT). This mutation is found in 5% to 10% of patients presenting with venous thrombosis and in approximately 15% of patients being investigated for thrombophilia [2]. Although controversial, it has also been associated with an increased risk of pregnancy loss, preeclampsia, intrauterine growth restriction, and placental abruption, among other complications [3,4]. PRT is produced by a substitution of a single base pair, replacing guanine with adenine, at the position of nucleotide 20,210 on chromosome 11p-q12, increasing the level of prothrombin (precursor of thrombin) and consequently the risk of venous thrombosis or pulmonary embolism [5,6]. Considering that PRT produces high levels of circulating prothrombin in the plasma, there is no phenotypic detection test, and detection is performed by genotyping by PCR (polymerase chain reaction) [7] or other assays [8,9,10]. A suitable alternative that does not require sample amplification and that has the potential to detect the mutation in a single base pair is the surface plasmon resonance (SPR) technique.

SPR is an optical technique based on the formation of an evanescent wave at the interface between a dielectric medium, typically either liquid or air, on a free-electron-rich metal, such as Au or Ag, and is highly sensitive to small changes in refractive index, measuring changes in the refractive index associated with recognition between the analyte and the immobilized receptor. SPR has stood out as a tool for the analysis of biomolecular interactions and is currently used in various areas, such as chemistry, biology, medicine, pharmacology, and environmental research [11,12,13,14,15,16]. Works related to the detection of prothrombin by SPR have been reported when prothrombin is part of the coagulation cascade [17]. SPR has also been used for the real-time detection of blood coagulation and platelet adhesion [18,19] and to investigate the coagulation of blood plasma in real time as a function of the concentrations of thromboplastin and heparin [20]. However, no studies have been performed on detecting thrombophilia detection by detecting the mutation of the prothrombin gene.

Detection using SPR seeks to improve sensitivity and selectivity compared to other techniques already reported, without false positives, and with reusable biosensors [21]. Additionally, if properly designed, SPR-based biosensors are highly selective, providing an analysis in brief stages, without extensive sample preparation protocols, label-free and in real time [22]. Labeling-based methods are often used to look for interactions between a target molecule and a bioreceptor, such as DNA probes, attached on a sensor surface. The target molecule is labeled before the interaction or in the step after the recognition event on the surface [23,24], as labeling is a noneconomical process that requires considerable time and can interrupt binding interactions, mainly with proteins [25]. Notably, the multiple types of interactions of nucleic acid chains have been used for the construction of SPR biosensors [13,26,27,28], among others [29,30,31]. Oligonucleotides generate highly specific bonds with proteins, other strands, or ions, through hydrogen bonds, base-stacking, and van der Waals or electrostatic interactions, depending on the sequence that makes up the structure [32,33,34,35,36]. Thus, through oligonucleotide hybridization, it is possible to differentiate complementary sequences from those with changes of one or more nitrogenous bases in their structure using SPR [13,37,38]. In addition, hybridization can be achieved and improved by adjusting variables such as concentration, temperature, buffer composition, and ionic strength, which in turn allows specificity against partially complementary or noncomplementary strands. In this sense, controlling the ionic strength of the buffer has a great influence on hybridization without leading to an excessive change in the refractive index, unlike hybridization suppressors [37].

In this work, a glass chip covered with an ultrathin (50 nm) gold film was modified with oligonucleotide strands and used as an SPR biosensor. This study aimed to build a specific biosensor for the detection of mutated genes responsible for PRT. Thus, using the corresponding complementary strand, we were able to detect both types of genes: mutated and normal. The constructed biosensors were selective, producing a clearly distinct response for the hybridization of complementary and noncomplementary strands in buffer solutions. Real-time, label-free detection was achieved with a simple protocol to prepare the biosensor.

## 2. Materials and Methods

### 2.1. Instrumentation

Biosensing surface construction and hybridization assays were performed using an SPR instrument, the Reichert Dual channel SPR 7500DC model (USA), with the autosampler system included. The data acquisition was performed using Integrated SPR Autolink from Reichert Technologies. All data were processed using the TraceDrawer 1.6.1 and OriginPro 8.0 software.

### 2.2. Materials and Reagents

For the construction of the biosensors, the following reagents and solvents were used: a glass chip covered with an ultrathin (50 nm) gold film and immersion oil (refraction index 1.5150) provided by Reichert Technologies (USA), 4-mercaptobenzoic acid (4MBA, 99%, 154.19 g/mol), ethanol p.a. (99.8%), *N*-(3-dimethylaminopropyl)-*N*′-ethylcarbodiimide hydrochloride (EDC, ≥98%, 191.70 g/mol), *N*-hydroxysuccinimide (NHS, 98%, 115.09 g/mol), and ethanolamine (≥98%, 61.08 g/mol), provided by Merck Group (Germany), and nitrogen gas provided by Linde Group (Chile).

For the running solutions, the following materials were used: ultrapure water (18 MWcm^−1^) obtained from a Milli-Q water system (Synergy UV equipment), sodium chloride (NaCl, 58.44 g/mol), tris(hydroxymethyl)aminomethane hydrochloride (Tris-HCl, molecular biology grade, 157.6 g/mol), ethylenediaminetetraacetic acid (EDTA, disodium salt dihydrate, molecular biology grade, 372.2 g/mol), and polyoxyethylene (20) sorbitan monolaurate solution (Tween^®^ 20 for synthesis), and sodium hydroxide (NaOH, 40.00 g/mol) provided by Merck Group (Germany). All solutions were filtered and degassed before use.

The oligonucleotides, aminated mutated thrombophilia (MT-A), complementary mutated thrombophilia (MT-C), aminated normal thrombophilia (NT-A), and complementary normal thrombophilia (NT-C), were provided by Integrated DNA Technologies (USA). The oligonucleotide samples were dissolved in Tris-EDTA buffer before use. Table 1 shows the nomenclature and sequences of the oligonucleotides used. Table 2 shows the molar mass of each oligonucleotide, in addition to the number of bases, GC (Guanine-Cytosine) content, melting temperature, and strongest folding.

### 2.3. Preparation of the Biosensing Surface

Two biosensors were constructed. In the first biosensor, called the MT biosensor, MT-A strands were immobilized to recognize MT-C strands in order to enable the detection of PRT. In the second, called the NT biosensor, NT-A strands were immobilized to recognize NT-C strands in order to detect the normal conditions for the coagulation process.

The MT and NT biosensors were constructed using the same protocols and conditions. First, the gold chip surface was modified with a self-assembled monolayer of 4MBA via drop coating outside the instrument: 150 μL of the solution (1.0 mM in ethanol) was placed in a closed petri dish for 30 min at room temperature. Then, the gold chip was washed with ethanol and dried using a stream of nitrogen gas. The 4MBA/gold chip was placed on a drop of immersion oil (7 μL) in the SPR instrument.

**Figure 1:**
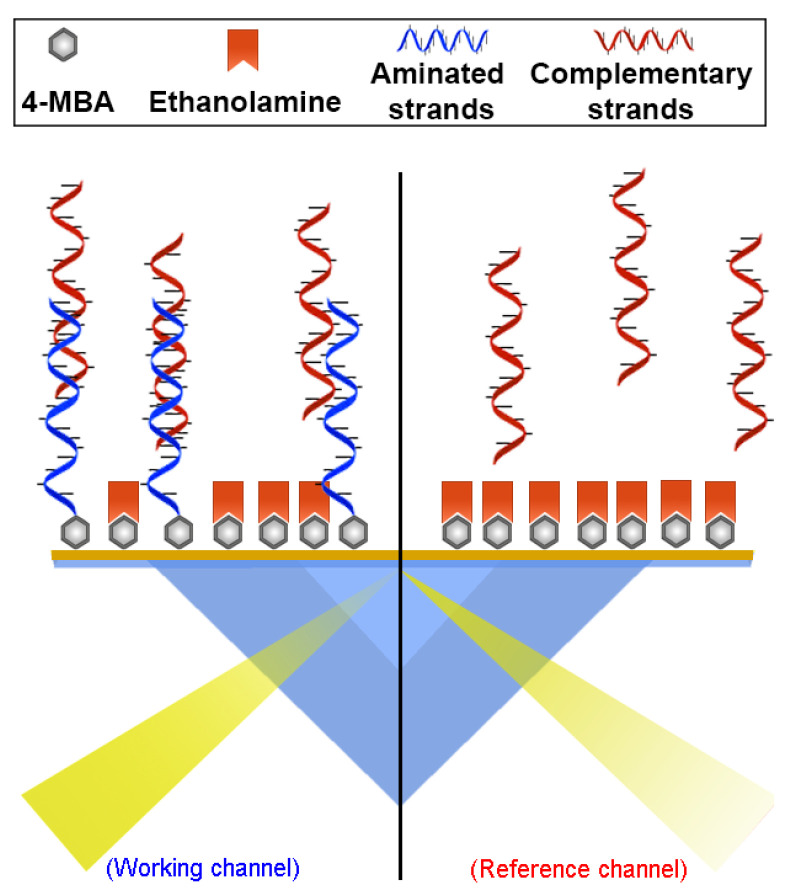
Schematic representation of the biosensor constructed, showing the working channel (**left side**), composed of a monolayer of 4-mercaptobenzoic acid, aminated thrombophilia strands, and ethanolamine, and the reference channel (**right side**), composed of 4-mercaptobenzoic acid and ethanolamine, and the interactions with complementary thrombophilia strands.

The SPR setup has two channels: the working and reference channels. In both channels, the carboxyl groups were activated with an EDC/NHS reaction. Aqueous solutions of EDC (0.4 M) and NHS (0.1 M) were prepared and mixed (in equal volumes) immediately before the experiments. Then, 500 μL of the EDC/NHS mixture (final concentration of 0.2 M/0.05 M) was injected twice at a 20 μL/min flow rate for 1500 s and the working channel was modified with the respective amino-oligonucleotides (MT-A or NT-A) using 250 μL (1.4 μM in Tris-EDTA) at a 5 μL/min flow rate for 3000 s, while the reference channel remained closed. Finally, 750 μL of ethanolamine (0.1 M to pH 8.0) was used three times at a 5 μL/min flow rate for 9000 s to block the free sites in both channels (more details are provided in the Supplementary Materials, Section 1 and Section 2). Figure 1 shows a scheme of the constructed biosensors.

### 2.4. Hybridization Assays

Complementary strands (MT-C or NT-C) were injected at different concentrations for 30 min at a flow rate of 5.0 μL/min (150 μL of each sample) using Tris-EDTA buffer as the run solution. The buffer was prepared with a mixture of Tris (10 mM), EDTA-HCl (1.0 mM), NaCl (0.3 M), and Tween^®^ 20 (0.25% *v*/*v*). After each hybridization assay, the biosensor surface was regenerated using HCl (10 mM) for 10 min at a flow rate of 20 μL/min (more details are provided in the Supplementary Materials, Section 3 and Section 4).

### 2.5. Data Processing Obtained by SPR

The association curves were corrected by double reference, discounting the response obtained from the reference channel and of an injected buffer solution.

The binding sites (Equation (1)) were obtained using the response of MT-A or NT-A in the immobilization stage, the surface density (Equation (2)), and its molecular weight. Complementary strand hybridization response graphs on each biosensor were fitted to a Langmuir isotherm model with one binding site (Equation (3)), obtaining KD and the maximum response, while Ka corresponds to the inverse of KD (Equation (4)). A linear adjustment was performed for the graphs of responses versus complementary strand concentration in each biosensor (Equation (5)). Sensitivity was obtained from the slope of the curve. In addition, the theoretical maximum response and ligand activity were calculated using Equations (6) and (7), respectively (more details are provided in the Supplementary Materials).

The limits of detection (LOD) and quantification (LOQ) were calculated experimentally for samples with final volumes of 150 μL, using as references the noise signal and the response generated by the buffer injection as a running solution.

(1) $$Binding\;site=\;\frac{Surface\;density}{Mw_{aminated\;strand}}\cdot 10,000\;\left(\mathrm{mol}\;\cdot {\mathrm{cm}}^{-2}\right)$$

(2) $$Surface\;density=\;\frac{R_{aminated\;strand}}{1,000,000}\;\left(g\;\cdot m^{-2}\right)$$

(3) $$y=\;\frac{R_{max}\cdot x}{KD+x}$$

(4) $$Ka=\;\frac{1}{KD}(M^{-1})$$

(5) y= Intercept+Slope·x

(6) $$Rmax_{theoretical}=\;\frac{Mw_{\begin{array}{c} complementary\;\\ strand \end{array}}\cdot R_{\begin{array}{c} aminated\\ strand \end{array}}\;\cdot Valency_{strand}}{Mw_{\begin{array}{c} aminated\\ strand \end{array}}}\;\left(RU\right)$$

(7) $$Ligand\;activity=\;\frac{R_{max}}{SPR\;response_{\begin{array}{c} aminated\\ strand \end{array}}}\;\cdot \frac{Mw_{\begin{array}{c} aminated\\ strand \end{array}}}{Mw_{\begin{array}{c} complementary\;\\ strand \end{array}}}\;\cdot 100$$

## 3. Results and Discussion

### 3.1. SPR Biosensor Construction

To improve the response of the MT and NT biosensors, the concentrations of the immobilized species on the surface of the gold chips, the flow rate, and the components in the run solution were evaluated (see details in Section 1, in the Supplementary Materials). A direct immobilization of the strands, by sorption or with thiol groups bound directly in its structure, can cause a structural change and blocking of the binding sites. In addition, a weak interaction with the surface generates an unstable and nonreproducible detection of the hybridization process. The recommended general strategy is to immobilize the probe by covalent bonds and a spacer molecule, which can be achieved using a self-assembled monolayer and EDC/NHS reaction [39].

The construction of each biosensor began by forming a self-assembled monolayer of 4MBA on the gold surface of the chip outside the equipment via drop coating, as has been previously reported [40,41]. Thus, the COOH groups of 4MBA remained exposed on the surface of the chip. Then, the chip was placed in the SPR equipment for the following modification steps.

**Figure 2:**
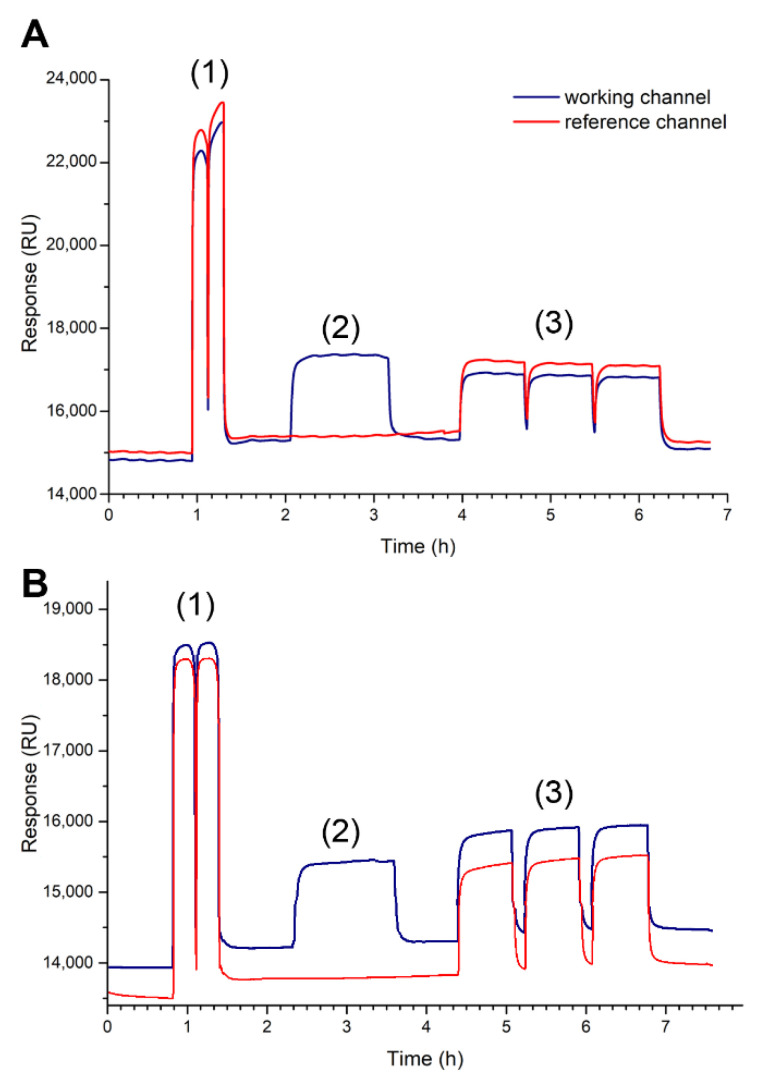
Sensorgrams of the construction process of the mutated thrombophilia (MT) biosensor (**A**) and normal thrombophilia (NT) biosensor (**B**). Sequentially, the observed responses correspond to (1) the activation stage using EDC/NHS; (2) the immobilization stage of the aminated thrombophilia strands (animated mutated thrombophilia (MT-A) and animated normal thrombophilia (NT-A), respectively); and finally (3) the blocking stage using ethanolamine.

Figure 2 shows the activation, immobilization, and blocking process, for the MT and NT biosensors. Activation of the COOH groups of 4MBA was performed using the EDC/NHS reaction, injected into both channels (working and reference), and the number of modified sites increased after a second injection, with an average change of 757 ± 20 RU (*n* = 10).

Subsequently, the reference channel was closed and a 1.4 μM solution of the amino-oligonucleotide (MT-A or NT-A) was injected into the working channel. The average change was 137 RU ± 10 (*n* = 10) for the MT biosensor, with a binding site area of 2.56 × 10^−12^ ± 1.87 × 10^−13^ mol/cm^2^. For the NT biosensor, the average change was 122 RU ± 11 (*n* = 10) with a binding site area of 2.26 × 10^−12^ ± 2.03 × 10^−13^ mol/cm^2^ (more details of calculations and values obtained in Section 2, in the Supplementary Materials). This demonstrates an efficient and nonsaturated immobilization of oligonucleotides on each surface, which could produce a higher degree of hybridization of its complementary strands [26,37,42].

Finally, ethanolamine was injected into both channels to block the still activated sites of the modified chip, and the injection was performed three times continuously to minimize nonspecific interactions.

### 3.2. Hybridization and Regeneration Processes

For the hybridization process, a running solution of Tris-EDTA buffer with Tween^®^ 20 at pH 7.5 was used. Additionally, NaCl was incorporated in the running solution to obtain conditions commonly used for the detection of oligonucleotides in SPR biosensors [26,43]. The increase in the ionic strength to 0.3 M was demonstrated to allow an increase in the hybridization response between complementary strands due to the shielding of negative charges of phosphate groups of the oligonucleotides. These conditions favor mainly hydrogen bonding interactions between the nucleic acids of the complementary bases [44,45].

**Figure 3:**
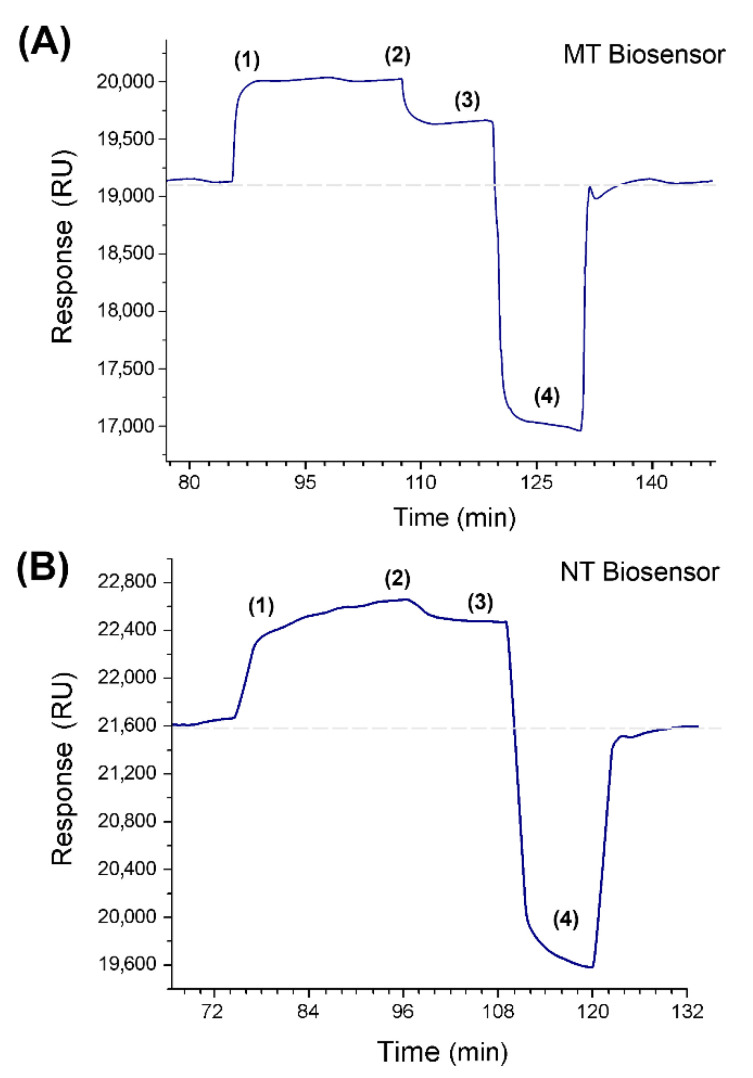
Response sensorgrams for a 40 nM solution of MT-C (**A**) and NT-C (**B**) injected into the respective biosensors, highlighting the different stages during the hybridization process of complementary strands. (1) Association, (2) equilibrium, (3) dissociation, and (4) regeneration.

Figure 3 shows the response of the hybridization process obtained using a 40 nM solution of complementary strands on (A) MT and (B) NT biosensors. The stages of association (1), equilibrium (2), dissociation (3), and regeneration (4) of each biosensor are highlighted and clearly identified. The association corresponds to the recognition stage and interaction, which increase with the injection time, reaching a state of equilibrium in which the association and dissociation of the strands occur at the same speed. The dissociation stage corresponds to the period in which only the buffer solution was injected, removing strands that were weakly retained by each biosensor and reaching a new base state. In our case, both association curves are exponential but differ in the rate of association and dissociation. In general, the MT-C injection shows an association curve that reaches equilibrium at 300 s, whereas the NT-C association takes 1200 s.

For the complete regeneration of the surface of each biosensor, a solution of HCl was added, according to that reported by Altintas et al., to dehybridize complementary strands of oligonucleotides, although in our case, the concentration was 10 times lower [43]. Each sensorgram returns to the baseline in an average of 600 s after this time probe, i.e., the immobilized amino-oligonucleotide is ready to start a new hybridization process through a new injection of complementary strands using the same biosensor. This process was repeated up to 24 times, and 95% of the initial response was maintained (see assays in Section 3, in the Supplementary Materials).

### 3.3. Analytical Performance of MT and NT Biosensors

Multiple injections of different concentrations of complementary strands were made into the MT and NT biosensors to study the variations in the refractive index. Each response in the association stage was obtained by applying a double correction, the response generated by nonspecific interactions in the reference channels, which had no immobilized amino-oligonucleotides (blank 1), and the response generated by the interaction of the buffer solution with the biosensors (blank 2). Therefore, the final variation obtained in each curve (*n* = 4) corresponds to a relationship between the interactions of the injected strands MT-C or NT-C and their respective immobilized strands of MT-A or NT-A (see Section 4, in the Supplementary Materials).

**Figure 4:**
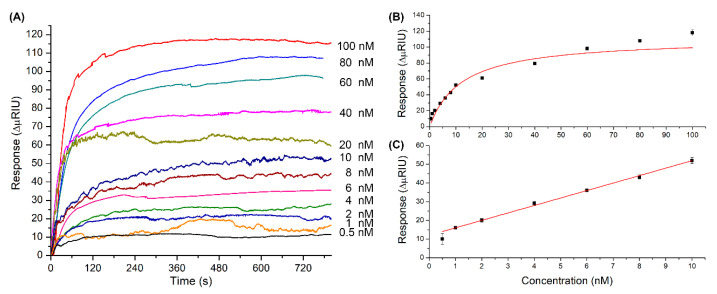
(**A**) Responses obtained for different concentrations of mutated thrombophilia (MT-C) injected into the MT biosensor (association stage); (**B**) graph of the responses obtained versus concentrations of MT-C injected; and (**C**) linear range evaluated from 0.5 and 10 nM (*n* = 4).

Figure 4 shows the response graphs of the MT biosensor. Figure 4A corresponds to the association curves obtained at different concentrations of MT-C from 0.5 to 100 nM at 25 °C and pH 7.5. Figure 4B corresponds to the plot of concentration versus response (*n* = 4), with increasing concentrations of MT-C; the response increases gradually and consistently to a Langmuir adsorption isotherm with one binding site [46,47]. A linear range was determined between 1.0 and 10 nM, with a correlation coefficient R^2^ of 0.99 (Figure 4C). The sensitivity of the system determined from the slope of the calibration curve was 4.0 ΔμRIU/nM (±0.1) (see calculations and values obtained in Table S3, in the Supplementary Materials).

**Figure 5:**
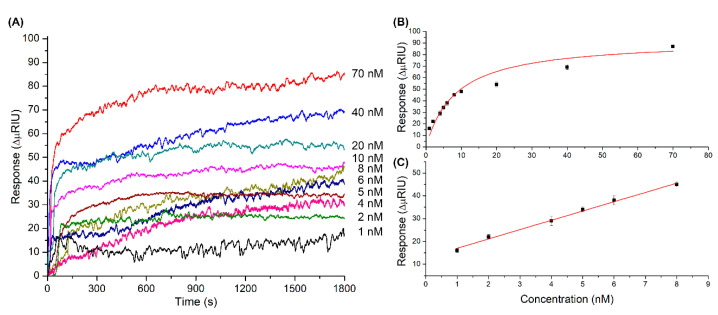
(**A**) Responses obtained for different concentrations of MT-C injected into the biosensor (association stage); (**B**) graph of the responses obtained versus concentrations of NT-C injected; and (**C**) linear range evaluated from 1.0 to 8.0 nM (*n* = 4).

Figure 5 shows the response graphs of the NT biosensor. The association curves obtained at 25 °C and pH 7.5 ranged from 1.0 to 100 nM (Figure 5A). The concentration versus response obtained was graphed (*n* = 4) (Figure 5B) and is also consistent with a Langmuir adsorption isotherm with one binding site [46,47]. The linear range was determined between 1.0 and 8.0 nM, with an adjustment of R^2^ of 0.99 (Figure 5C). The sensitivity of the system determined from the slope of the calibration curve was 4.1 ΔμRIU/nM (±0.1) (see calculations and values obtained in Table S4, in the Supplementary Materials).

The mathematical adjustments described in Section 2.5 were applied only to study the hybridization between complementary strands, that is, the MT biosensor with MT-C and the NT biosensor with NT-C. The dissociation constant (KD) obtained was 12.2 nM (±1.8) for the MT biosensor and 8.5 nM (±1.2) for the NT biosensor. The data show that both biosensors have similar affinities for their complementary strands, and their values are consistent with those reported in the literature [47,48]. Additionally, the binding capacity between complementary oligonucleotides can be evaluated through the ligand activity. The activity of the MT biosensor was 84% (±6) and that of the NT biosensor was 80% (±4), which is explained by the maximum responses observed during the respective hybridization processes, which are slightly less than the theoretical maximum responses calculated. These results were expected, considering that factors such as the loads and disposition of the strands, in addition to the time and speed of binding, among other factors, prevent the immobilized oligonucleotides from being 100% active. It is necessary to highlight that the maximum response and the activity of each biosensor do not vary significantly between tests, confirming that the surface can be reused up to 24 times as previously discussed (see calculations and values obtained in Table S5, in the Supplementary Materials).

For the detection of MT-C in the MT biosensor, the LOD was 20 pM (3.0 fmol) and the LOQ was 70 pM (10.5 fmol). For the detection of NT-C in the NT biosensor, the LOD was 30 pM (4.5 fmol) and the LOQ was 110 pM (16.5 fmol). The LOD and LOQ reported in this investigation are of the same orders of magnitude or lower than those in studies of the detection of other analytes using SPR technology through the hybridization of oligonucleotides of complementary sequences of similar size and that have not incorporated nanomaterials to improve the response obtained [11,43,49]. In the specific field of PRT detection, there is no method equivalent to the one proposed in this investigation. PCR-based methods, which apply protocols to amplify the concentration of the analyte, were excluded. Cooper and Rezender argue that assays that are not based on PCR have the disadvantage of requiring relatively high concentrations, >10 ng/μL, of the sequence in the sample [50]. In our case, the developed biosensors detected concentrations lower than 10 ng/μL of the mutated strand in PRT disease.

### 3.4. Interchip Reproducibility Study

**Figure 6:**
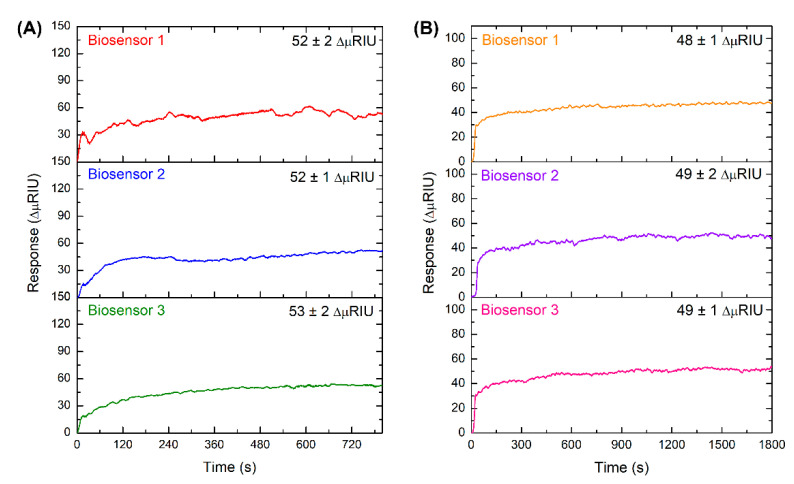
Detection responses of MT-C (**A**) and NT-C (**B**) strands at 10 nM on the three biosensors constructed.

Three MT and NT biosensors were constructed to demonstrate the reproducibility of the detection of complementary strands. For this purpose, MT-C or NT-C strands at a 10 nM concentration were injected into each biosensor five times, and the average responses were obtained at equilibrium, which ranged from 240 to 780 s and 900 to 1800 s, respectively. Figure 6 shows the sensorgrams of MT (A) and NT (B) biosensors, which are reproducible in their association profiles and maximum responses obtained.

### 3.5. Selectivity Assays of Complementary versus Noncomplementary Strands

**Figure 7:**
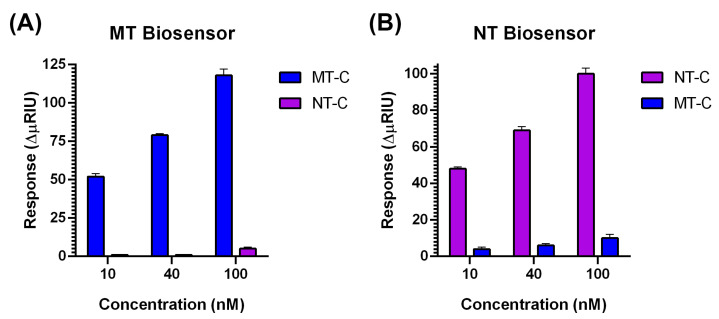
(**A**) Graph of the response of complementary (MT-C) versus noncomplementary strands (NT-C) in the MT biosensor and (**B**) graph of the response of complementary (NT-C) versus noncomplementary strands (MT-C) in the NT biosensor. The concentrations studied were 10, 40, and 100 nM, with *n* = 4.

To evaluate the specificity of hybridization of MT and NT biosensors, assays were carried out with the complementary strands and the noncomplementary counterpart, i.e., MT-C and NT-C, with both biosensors. Figure 7 shows the graphs of responses obtained for both biosensors at concentrations of 10, 40, and 100 nM MT-C and NT-C.

The complementarity between the amino-oligonucleotide (MT-A and NT-A) immobilized in each biosensor with its respective complementary strands (MT-C and NT-C) is given by interactions between their nucleobases, which are held together mainly by hydrogen bonding. This bonding is highly efficient between adenine and thymine and between guanine and cytosine. The mutation present in the thrombophilia strands corresponds to a change in nucleobases from guanine to adenine. The GC content varies from 58.8% for NT-C to 52.9% for MT-C. These differences cause a decrease in interactions and even changes in secondary structure, which was also revealed experimentally with the calculated KD. Therefore, the change in one base pair hinders hybridization between normal and mutated thrombophilia strands because they are not exactly complementary.

In the MT biosensor, with MT-A strands immobilized, injections of the same concentration of both sequences (MT-C and NT-C) showed a clear difference between SPR responses. The responses in association with MT-C at the three concentrations studied were 118 ± 4, 79 ±1 and 52 ± 2 ΔμRIU, while all NT-C injections were found at the noise level (Figure 7A). This demonstrates that the hybridization of the mutated strand MT-A is highly selective for MT-C, while NT-C hybridization is null.

For the NT biosensor with NT-A immobilized, larger numbers of interactions with both strands are expected due to the higher percentage of GC content. In the assays at 10, 40, and 100 nM, the injections of NT-C showed consistently higher responses than MT-C injections (Figure 7B). Specifically, at a concentration of 100 nM, the interaction between MT-C and the NT biosensor showed a response of 10 ± 2 ΔμRIU, which is 10 times lower than the NT-C response (100 ± 3 ΔμRIU). Even at the highest concentration studied, the NT biosensor allows selective detection between complementary (NT-C) and noncomplementary (MT-C) strands.

## 4. Conclusions

The results of this study show that the MT biosensor generated can be used to detect the mutated gene of PRT (one pair base of difference with the normal one) with high selectivity. Analogously, the NT biosensor is highly selective for the detection of NT-C strands against MT-C strands, and can be used as a control. The responses were obtained in real time in a process that takes 30 min for label-free strand detection and without requiring an amplification method. In addition, both biosensors are reusable for the injection of new samples when the surface is regenerated with HCl.

To our knowledge, this is the first work that focuses on the detection of prothrombin-related thrombophilia by analyzing the mutated and non-mutated section of DNA and that does not require amplification of the sample. In this sense, it is a significant advance in the detection of single point mutations using a simple SPR protocol, with high selectivity and with limits of detection and sensitivity in the same range as those reported by other methods. New studies must be carried out to determine selectivity between mutated and non-mutated strands in biologically relevant media and real samples.

The incorporation of graphene-derived materials on the sensing surface can improve the sensitivity and the detection limits, allowing them to reach concentrations between fM to aM. This would avoid the extraction of cellular DNA and the exhaustive treatments of the sample such as accumulation or labeling, since the analysis could be carried out on ultra-low concentration samples such as circulating cell-free DNA extracted from saliva or urine, whose method of obtaining is rapid and minimally invasive.

## Figures and Tables

**Table 1:** Sequences of oligonucleotides used.

Oligonucleotide Type	Code	Sequence
Mutated Probe	MT-A	5’-/NH_2_(CH_2_)_6_/CAT TGA GGC TTG CTG AG-3’
Mutated Target	MT-C	5’-CTC AGC AAG CCT CAA TG-3’
Normal Probe	NT-A	5’-/NH_2_(CH_2_)_6_/CAT TGA GGC TCG CTG AG-3’
Normal Target	NT-C	5’-CTC AGC GAG CCT CAA TG-3’

**Table 2:** Data on the oligonucleotides used.

Oligonucleotide Code	Molecular Weight	DNA Bases	GC Content	Tm (50 mM NaCl)	Strongest Folding Tm
MT-A	5340.4 g/mol	17	52.9%	50.8 °C	23.2 °C
MT-C	5139.4 g/mol	17	52.9%	50.8 °C	−16.4 °C
NT-A	5405.6 g/mol	17	58.8%	53.2 °C	24.2 °C
NT-C	5155.4 g/mol	17	58.8%	53.2 °C	10.6 °C

## Supplementary Materials

The following are available online at https://www.mdpi.com/1424-8220/20/21/6240/s1, Figure S1: Sensorgram of injections of the MT-C strands (40 nM) into the working channel of the MT biosensor, Figure S2: Sensorgram of injections of the TN-C strands (40 nM) into the working channel of the NT biosensor, Table S1: Responses, surface density and binding sites calculated for the immobilization of MT-A and NT-A on MT and NT biosensors, respectively, Table S2: Responses obtained for the hybridization process between complementary strands at different concentrations, Table S3: Equations and values of the mathematical adjustments applied in the response versus concentration graph of MT-C injected into the MT biosensor, Table S4: Equations and values of the mathematical adjustments applied in the response versus concentration graph of NT-C injected into the NT biosensor, Table S5: Maximum responses, constants and ligand activity obtained for the MT and NT biosensors.
