# Supplementary material for: Label-Free Oligonucleotide-Based SPR Biosensor for the Detection of the Gene Mutation Causing Prothrombin-Related Thrombophilia
Source: Sensors (Basel). 2020 Oct 31;20(21):6240. doi: 10.3390/s20216240 (PMC7663036; doi:10.3390/s20216240)
Supplement: Supplementary file 1 [file sensors-20-06240-s001.pdf]

sensors

MDPI

Electronic Supplementary Material (ESM)

Label-free oligonucleotide-based SPR biosensor for the detection of the gene mutation causing prothrombin-related thrombophilia

Rodrigo Sierpe[1,2,3], Marcelo J. Kogan[2,3] and Soledad Bollo*[1,3]

[1]Laboratorio de Biosensores, Facultad de Ciencias Químicas y Farmacéuticas, Universidad de Chile. Santos Dumont N° 964, Independencia, Santiago-Chile.
[2]Laboratorio de Nanobiotecnología y Nanotoxicología, Facultad de Ciencias Químicas y Farmacéuticas, Universidad de Chile. Santos Dumont N° 964, Independencia, Santiago-Chile.
[3]Advanced Center for Chronic Diseases (ACCDiS), Universidad de Chile and Pontificia Universidad Católica de Chile, Santiago, Chile.

S1 Construction of biosensors with mutated or normal thrombophilia strands

To improve the response of each biosensor, all stages of modification of the gold surface were optimized.

Self-assembled monolayer formation: 150 L of 4MBA solution in ethanol was evaluated at 10 and 1.0 mM. The concentration of 4 MBA (1.0 mM) was chosen due to the greater reproducibility in the responses obtained for the activation stage using EDC/NHS.

Activation of carboxylic groups: One or two injections of EDC/NHS (0.2 M and 0.05 M, respectively) were evaluated. Two injections of EDC/NHS were used since the first and second injections both registered increases in the activation response of 545 ± 14 RU and 212 ± 25 RU, respectively (n =10). Consequently, using two injections, a greater response was recorded in the immobilization of aminated strands.

Strand immobilization: Two types of buffer solutions were evaluated, PBS and Tris-EDTA, at pH 7.4, with flow rates of 5, 10 and 20 L/min, ionic strength of 0.1 or 0.3 M, and Tween20 0.25% v/v. At a 1.4 M concentration of MT-A or NT-A strands, Tris-EDTA buffer was used at 5 L/min for 50 min, without ionic strength and without Tween20. The selection criterion was the highest response obtained due to the greatest number of immobilized strands, with 137 ± 10 RU and 122 ± 11 RU for the immobilization of MT-A and NT-A, respectively (n =10).

Blocking: An injection of 1.0 M ethanolamine was compared with successive injections (between 1 and 5) of 0.1 M ethanolamine at pH 8.5, adjusted with HCl. Three consecutive injections of ethanolamine 0.1 M at pH 8.5 were used due to the higher response obtained in the working channel and lower response in the reference channel during the hybridization stage with complementary strands MT-C or NT-C.

S2 Binding site calculations

All data were obtained considering the molecular weights of aminated and complementary strands described in Table 2, Section 2.2 of the Materials and Methods. Table S1 shows the immobilization response of MT-A or NT-A and the surface density and binding sites calculated using the respective equations.

$$Binding\ site = \frac{Surface\ density}{Mw_{aminated\ strand}} \times (mol\ \times cm^{-2}) \qquad (1)$$

$$Surface\ density = \frac{R_{aminated\ strand}}{1000000}\ (g\ \times\ m^{-2}) \qquad (2)$$

Table S1. Responses, surface density and binding sites calculated for the immobilization of MT-A and NT-A on MT and NT biosensors, respectively.

	Response Aminated Strand (RU)	Surface Density (g/cm²)	Binding Site (mol/cm²)
MT Biosensor	137 ± 10	$1.37\times10^{-8} \pm 1.0\times10^{-9}$	$2.56\times10^{-4} \pm 1.87\times10^{-5}$
NT Biosensor	122 ± 11	$1.22\times10^{-8} \pm 1.1\times10^{-9}$	$2.26\times10^{-4} \pm 2.03\times10^{-5}$

S3 Reuse of each biosensor

Figure S1 and S2 show 24 consecutive injections of complementary strands at a concentration of 40 nM into the corresponding biosensor, maintaining over 95% of the initial response.

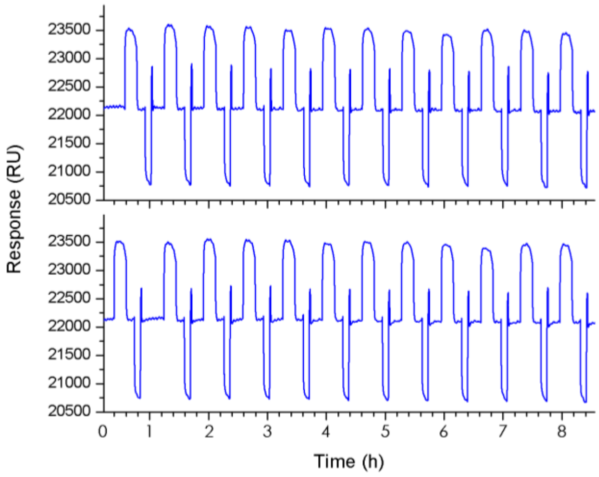

Figure S1. Sensorgram of injections of the MT-C strands (40 nM) into the working channel of the MT biosensor.

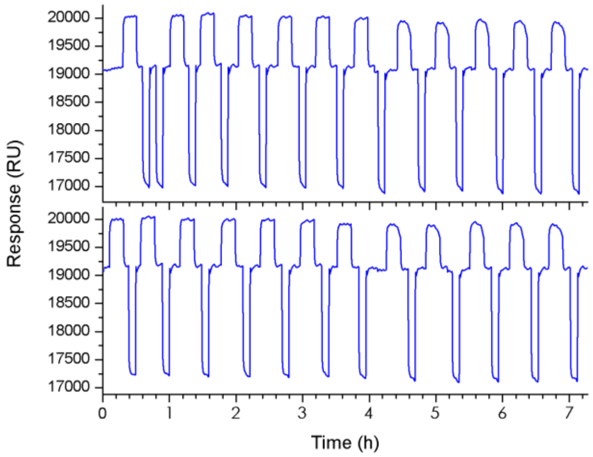

Figure S2. Sensorgram of injections of the TN-C strands (40 nM) into the working channel of the NT biosensor.

S4 Hybridization assays between complementary strands

Table S2 shows the concentrations, average responses and standard deviations obtained for injections of complementary strands MT-C (in the MT biosensor) and NT-C (in the NT biosensor).

Table S2. Responses obtained for the hybridization process between complementary strands at different concentrations.

**MT Biosensor**			**NT Biosensor**		
**MT-C Concentration (nM)**	**Response (Δ RIU) (n=4)**	**Standard Deviation**	**NT-C Concentration (nM)**	**Response (Δ RIU) (n=4)**	**Standard Deviation**
100	118	4	100	100	3
80	108	2	70	87	1
60	98	2	40	69	2
40	79	1	20	54	2
20	61	1	10	48	1
10	52	2	8.0	45	1
8.0	43	1	6.0	38	2
6.0	36	0.4	5.0	34	1
4.0	29	1	4.0	29	2
2.0	20	1	2.0	22	1
1.0	16	0.6	1.0	16	1
0.5	10	3			

S5 Mathematical adjustments for the curves of response versus concentration

Table S3 shows the equations used and parameters obtained for the response versus concentration of complementary strands in the MT biosensor. The first adjustment was a Langmuir isotherm model for the responses obtained from MT-C between 0.5 and 100 nM. Then, a linear fit was used in the range between 0.1 and 1.0 nM.

Table S3. Equations and values of the mathematical adjustments applied in the response versus concentration graph of MT-C injected into the MT biosensor.

Equation	$\boldsymbol{y = \frac{R_{max} \times x}{KD + x}}$	R-square = 0.94702
Parameter	Value	Standard error
R*max*	111.4206	7.8850
KD	12.2018	1.8586
Equation	$\boldsymbol{y = Intercept + Slope \times x}$	R-square = 0.99675
Parameter	Value	Standard error
Intercept	12.0044	0.4813
Slope	3.9961	0.0932

Table S4 shows the equations used and parameters obtained for the response versus concentration of complementary strands in the NT biosensor. The first adjustment was a Langmuir isotherm model for the responses obtained from NT-C between 0.1 and 10 nM. Then, a linear fit was used in the range between 0.1 and 0.8 nM.

Table S4. Equations and values of the mathematical adjustments applied in the response versus concentration graph of NT-C injected into the NT biosensor.

Equation	$y = \frac{R_{max} \times x}{KD + x}$	R-square = 0.95928
Parameter	Value	Standard error
R*max*	92.9441	5.0867
KD	8.4852	1.2413
Equation	$y = Intercept + Slope \times x$	R-square = 0.99342
Parameter	Value	Standard error
Intercept	12.9641	0.7232
Slope	4.0763	0.1483

Table S5 shows the values obtained for KD and Ka and the theoretical response maximum and ligand activity for both biosensors using the following equations:

$$Ka = \frac{1}{KD} \quad (M^{-1}) \tag{3}$$

$$Rmax_{theoretical} = \frac{Mw_{\substack{complementary \\ strand}} \times R_{\substack{aminated \\ strand}} \times Valency_{strand}}{Mw_{\substack{aminated \\ strand}}} \quad (RU) \tag{4}$$

$$Ligand\ activity = \frac{R_{max}}{SPR\ response_{\substack{aminated \\ strand}}} \times \frac{Mw_{\substack{aminated \\ strand}}}{Mw_{\substack{complementary \\ strand}}} \times 100 \tag{5}$$

Table S5. Maximum responses, constants and ligand activity obtained for the MT and NT biosensors.

	**Response Maximum (RIU)**	**KD (nM)**	**Ka (M^{-1})**	**Valency**	**Response Maximum (theoretical) (RIU)**	**Ligand Activity (%)**
**MT Biosensor**	111.4 ± 7.9	12.2 (± 1.8)	8.2×10^{7} ± 1.2×10^{7}	1:1	131.8 ± 9.6	84.5 ± 6.0
**NT Biosensor**	92.9 ± 5.1	8.5 (± 1.2)	1.2×10^{8} ± 1.7×10^{7}	1:1	116.4 ± 10.5	80.0 ± 4.4
